# Investigating seafood substitution problems and consequences in Taiwan using molecular barcoding and deep microbiome profiling

**DOI:** 10.1038/s41598-020-79070-y

**Published:** 2020-12-15

**Authors:** Pei-Ying Chen, Cheng-Wei Ho, An-Chi Chen, Ching-Yi Huang, Tsung-Yun Liu, Kung-Hao Liang

**Affiliations:** 1 Institute of Food Safety and Health Risk Assessment, National Yang-Ming University, No. 155, Section 2, Linong St, Beitou District, Taipei City, 112 Taiwan; 2 Department of Health, Taipei City Government, Taipei, Taiwan; 3 Department of Medical Research, Taipei Veterans General Hospital, Taipei, Taiwan; 4 Institute of Biomedical Informatics, National Yang-Ming University, Taipei, Taiwan

**Keywords:** Metagenomics, Bioinformatics

## Abstract

Seafood is commonly seen in cuisines of the Asia–Pacific regions. The rates and consequences of seafood substitution frauds in Taiwan were elusive. To address this, we conducted a consumer-centered study, collecting seafood dishes and cooking materials from restaurants and markets easily accessible to the residents in Taiwan. Seafood substitutions were evaluated using DNA barcodes in the mitochondrial MT-CO1 gene. Among the 127 samples collected, 24 samples were mislabeled (18.9%, 95% Confidence interval [CI] = [12.5–26.8%]). The mislabel rates vary in different fish and product types (snapper [84.6%, 54.6–98.1%], cod [25%, 5.5–57.2%], swordfish [16.7%, 2.1–48.4%], cobia [16.7%, 0.4–64.1%], surimi products [100.0%]). A deep microbiome profiling was performed in 8 correctly-labeled conventional sushi and 2 tilapia sashimi mislabeled as snapper, with sequencing depths greater than 100,000 reads for every sample. The relative abundance of *Pseudomonas* genus is significantly higher in tilapia sashimi than in conventional sushi (*P* = 0.044). In conclusion, the gross seafood mislabel rate in Taiwan is 18.9% (12.5–26.8%). Snapper, cod and surimi products are particularly vulnerable to fraudulent substitutions. The high abundance of *Pseudomonas* in tilapia sashimi mislabeled as snapper unveils a potential health issue pertaining to the consumption of raw mislabeled seafood.

## Introduction

**Figure 1:**
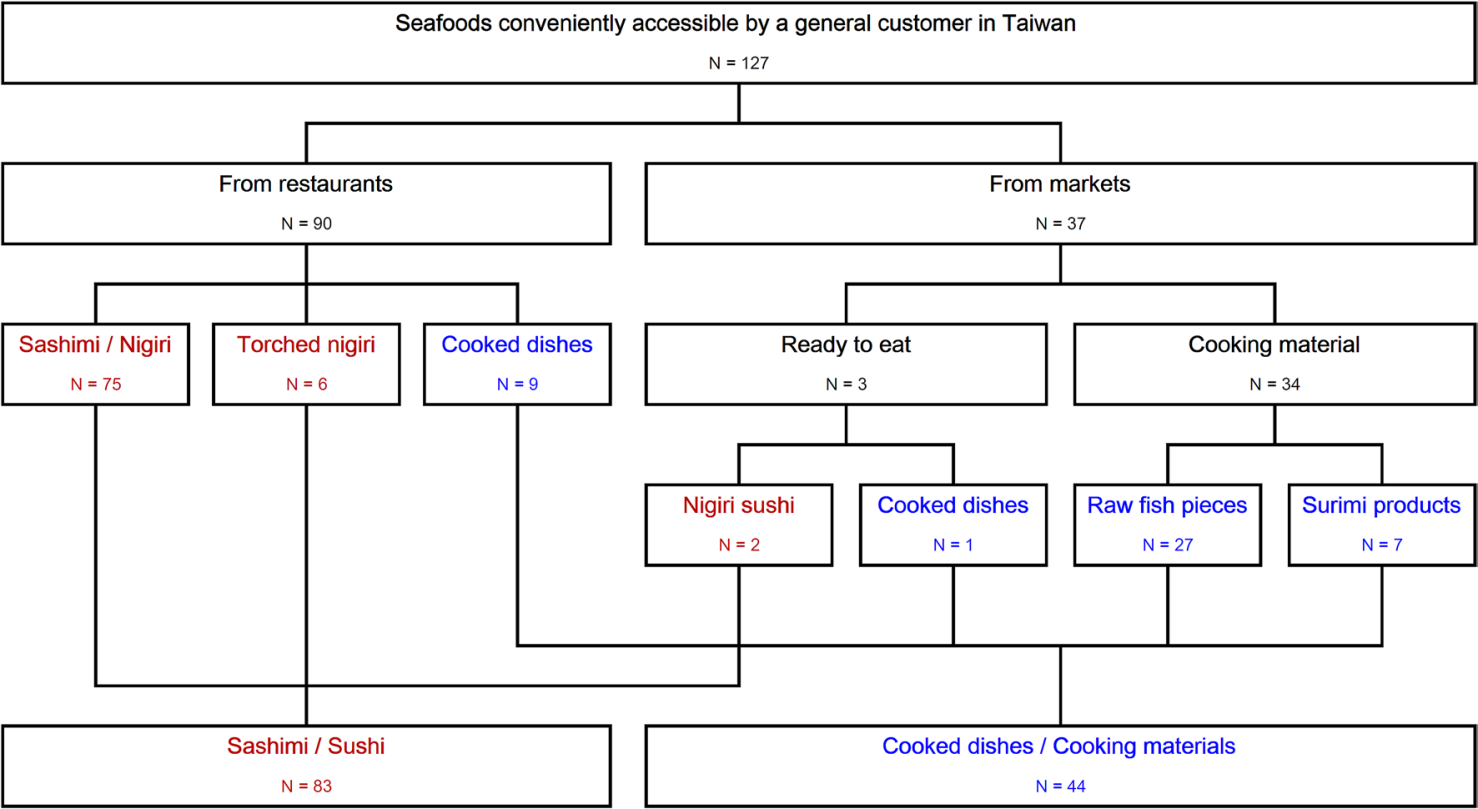
A schematic overview of the study. N: sample size.

Seafood and fresh-water fishes are commonly seem in the long human culinary history^1^, particularly in coastal regions such as Taiwan and Asia–Pacific regions. Seafood are easily accessible to the residents in Taiwan, in the form of raw cooking materials sold in retail markets and ready-to-eat meals served in restaurants and food stands (Fig. 1). Food markets and restaurants alike, correct product labels are essential for ensuring fair trades and preventing consumers from receiving pathogenic, allergenic or toxic seafood^2,3^. Product labels play the important role of summarizing and transmitting information along the long supply chain from fisheries to the consumers. However, these labels are subject to fraud^4^. Seafood substitution is a fraud in which the fish is sold by the name of a different, often more expensive fish^3^. Alert customers can verify the label correctness using the appearance (such as shapes and colors) and textures of the products. However, modern seafood products often appear in the form of fish pieces, filets or even ready-to-eat meals instead of whole fishes. The food processing technologies can also change the original color and luster of the meat. The lack of identifiable features poses challenges in verifying label information by the look, touch and feel of the products^5^.

The molecular barcoding technology is useful for revealing the true biological identity of the food, thereby discouraging food substitution misconducts^6^. The mitochondrial Deoxyribonucleic acid (DNA) has been used as reliable molecular barcodes for taxonomic identifications of a wide diversity of biological species^6–8^. The mitochondrial DNA harbors adequate amounts of single or short nucleotide variations, particularly in the regions encoding the cytochrome c oxidase subunit I (MT-CO1) gene and the 16S ribosomal RNA (16S rRNA) gene, facilitating the taxonomic identification. Recently, mitochondrial DNA is also widely used for deep microbiome profiling^9^.

**Figure 2:**
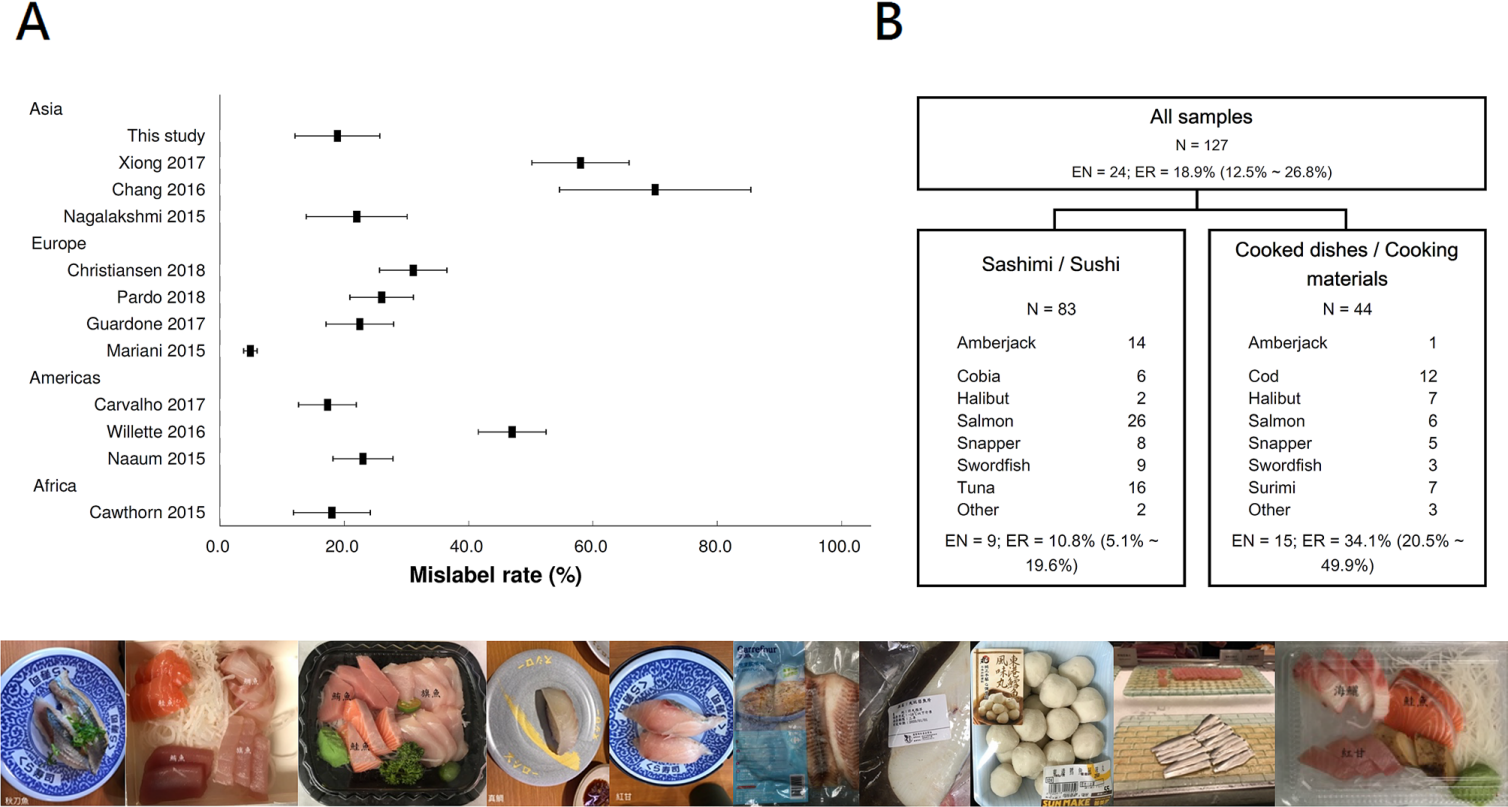
(**A**) The mislabel rate and 95% confidence interval in this study and in literature. (**B**) Seafood product categories and their corresponding mislabel rates in this study. A total of 127 seafood samples were collected. Among them, 83 samples are sashimi or sushi (containing fish meat served raw). 44 samples are either already cooked dishes or cooking materials acquired from markets. The gross mislabel rate is 18.9% (95% Confidence interval [CI] = [12.5–26.8%]). The “Other” categories comprise a Dotted gizzard shad sashimi and a Mackerel sashimi, as well as one Saury, one Mackerel, and one Tilapia labeled as “Taiwan Snapper” clearly distinguishable from conventional snapper. N: sample size. EN: Error number. ER: Error rate.

Using the molecular barcoding technology, the seafood substitution rates in different regions of the globe were gradually revealed. The substitution problem is inhomogeneous with respect to regions, fish types and product types. Snappers are frequently substituted by rockfish and tilapia in Canada^10^. Bluefin tuna (*Thunnus thynnus*) is often substituted by yellowfin and bigeye tuna (*T. albacares* and *T.obesus*) in Brussel’s restaurants and canteens^3^. Cod and sole are also frequently substituted^3^. The gross mislabel rate in supermarkets and fishmongers in Germany is 9.3%^11^. It is 23% in the supermarkets of Canada^12^, 42.8% in the fish fillets in southern Italy^13^ and 58% in the Cod products in China^14^. Ocean Canada reported that snapper, yellowtail, tuna, halibut, cod and salmon are fished types frequently substituted^10^. The substitution rates obtained in different regions are summarized in Fig. 2A, and the differences in which justify more investigations conducted from local consumers’ perspective.

Taiwan is an Asian island located at the gateway between East and Southeast Asia. The nearby countries include Japan, Korea, China, Vietnam, Malaysia, Singapore, and the Philippines. International trades have been popular. Taiwan is a popular tourist destination partly due to its reputations of cuisines. Fishing is an important industry in Taiwan, where ~ 100,000 households depend on fishing as the major economic source^15^. One million tons of seafood products are produced annually^15^. Residents in Taiwan consume 6–10 g of fish protein in average, ^16^ contributing more than 20% of all animal proteins received daily^16^. Depending on the cuisine, seafood are either consumed raw or with different degrees of cooking. Sashimi (fish meat pieces served raw), nigiri sushi (sashimi served on top of cooked, vinegar-flavored rice) and torched nigiri sushi (a nigiri sushi where the surface of the fish meat is slightly cooked by kitchen torches for adding a char flavor. The internal part remains raw) are internationally recognized Japanese cuisines. Taiwan is a previous Japanese colony between 1895 and 1945. As a result, Sashimi and sushi are widely accessible in high-end restaurants, average-priced chain restaurants and food stands in Taiwan. Tuna, swordfish, snapper, amberjack, and salmon are typical fish types used for sashimi and nigiri sushi. As sashimi are consumed raw, the health aspects are particularly critical.

Despite the importance of seafood in Taiwan, general customers’ exposure to mislabeled seafood remained unclear. A previous study in Taiwan showed a high mislabel rate of 70% among suspicious merchants picked up by the coastal petrol and custom officers^17^. We were thus motivated to conduct a consumer-centered survey in the domestic market of Taiwan.

## Results

### Assessment of seafood mislabel rate

A total of 127 seafood samples were collected from restaurants and markets in Taiwan between February 2018 and October 2020. Among them, 83 sashimi/sushi samples were collected from places such as food stands, normal-priced restaurants and high-end restaurants (Fig. 1). Additionally, 44 cooked meals or cooking-materials, including raw fish pieces, filets and surimi products, were collected from a variety of hypermarkets, supermarkets, traditional markets, seafood wholesaler/retailers and fishing harbors (Fig. 1).

Each sample was examined using DNA barcoding technology. Among the 127 samples, 24 samples were found substituted. The gross substitution rate is 18.9% (95% Confidence interval [CI] = [12.5–26.8%]), comparable to prior seafood substitution studies in Asia^18^, Europe^3,19,20^, Americas^12,21,22^ and Africa^23^ (Fig. 2**)**. It is, however, lower than the mislabel rate of suspicious merchandizes found in the customs and coastal petrol offices of Taiwan (Chang et al., 70%, *P* < 0.001)^17^. It is also lower than the mislabel rate of roasted Cod products investigated in China (Xiong et al., 58%, *P* < 0.001)^14^. It is also lower than the gross mislabel rate of sushi restaurants in Los Angeles, USA (Willette et al., 47%, *P* < 0.001, Fig. 2A**)**^21^. It is higher than the gross mislabel rate found in a pan-European study (Mariani et al., 4.9%, *P* < 0.001)^24^.

**Figure 3:**
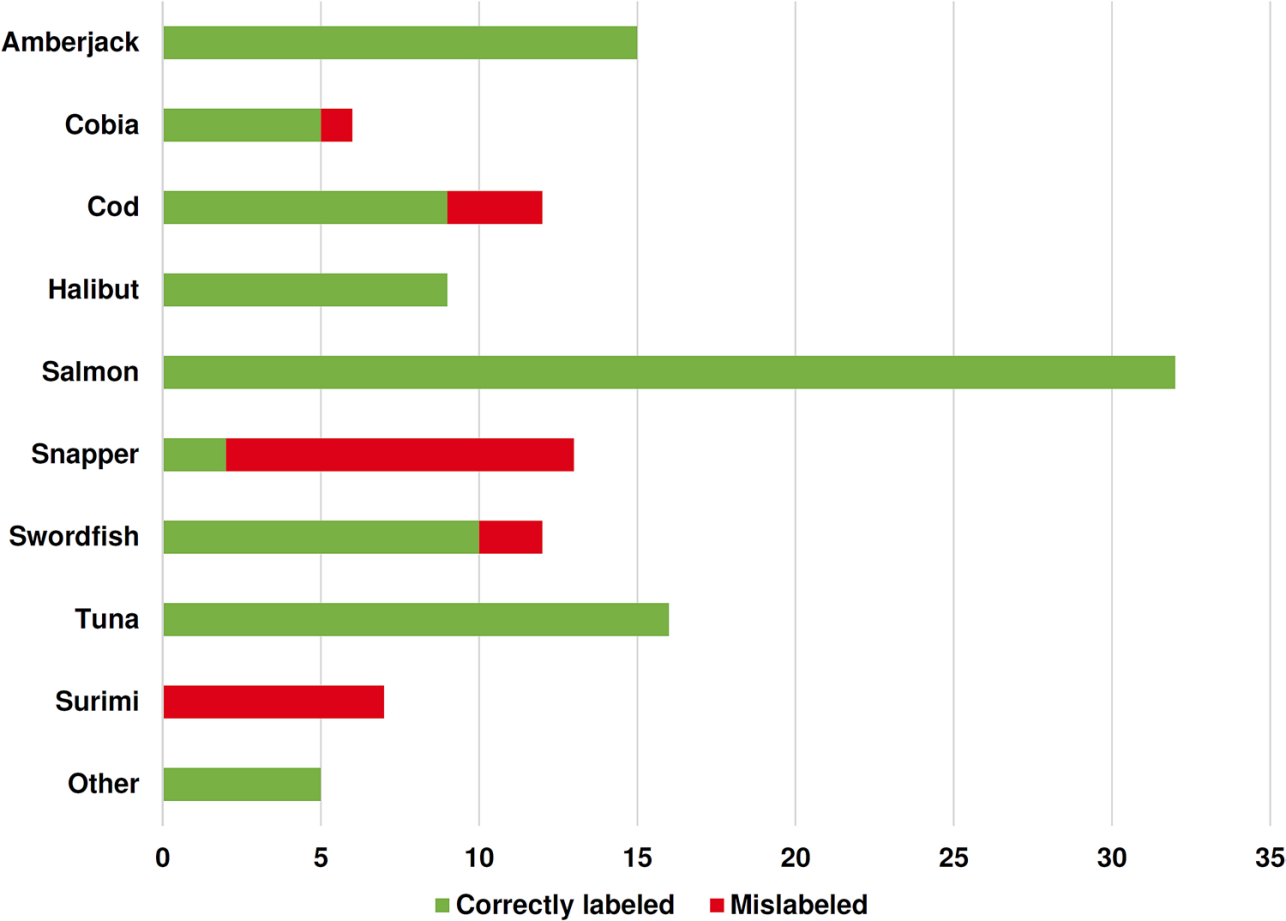
The correctly labeled and mislabeled seafood samples in this study.

The substitution events are not homogeneously distributed (Fisher’s exact *P* < 0.001, Fig. 3). Rather, they concentrated in certain categories including snapper (84.6%, 54.6–98.1%, 11/13), cod (25%, 5.5–57.2%, 3/12), swordfish (16.7%, 2.1–48.4%, 2/12), cobia (16.7%, 0.4–64.1%, 1/6) and surimi products (100.0%, 7/7). The substituted snappers are the largest mislabel category, where all 11 substituted snappers are molecularly confirmed to be tilapia (*Oreochromis niloticus,* Table 1). Additionally, one swordfish is substituted by atalntic salmon (*Salmo salar*). One other swordfish and one cobia are substituted by amberjack (*Seriola dumerili*). Three cod products are substituted by Greenland halibuts (*Reinhardtius hippoglossoides*).

**Table 1:** Mislabel events tabulated by barcode-identified fish species and product labels (not counting surimi products).

Barcode identified species	Labeled					Subtotal
	Sashimi and sushi (raw fish dishes)			Cooked dishes/cooking materials		
	Cobia	Snapper	Swordfish	Cod	Snapper	
*Oreochromis niloticus*	0	6	0	0	5	11
*Salmo salar*	0	0	1	0	0	1
*Seriola dumerili*	1	0	1	0	0	2
*Reinhardtius hippoglossoides*	0	0	0	3	0	3

### Deep microbiome profiling of tilapia and commonly consumed sashimi in restaurants

**Figure 4:**
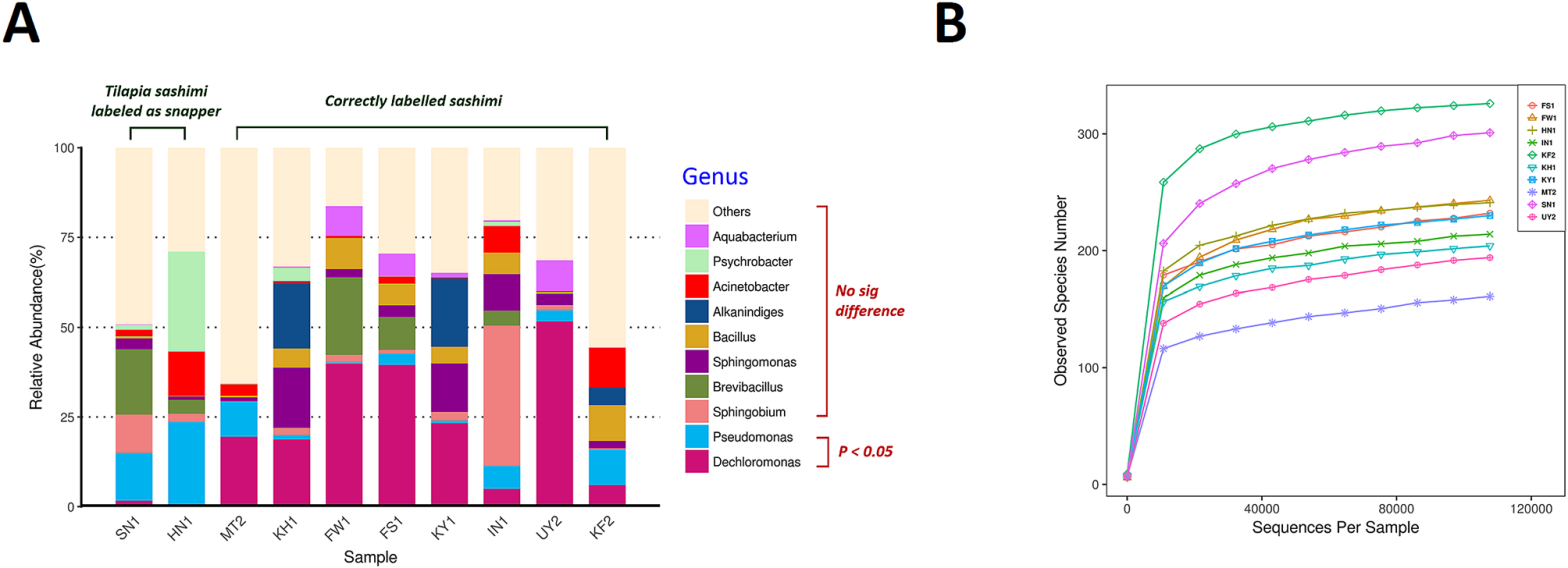
The microbiome analysis of tilapia and commonly-consumed sashimi samples in restaurants. (**A**) The relative abundance of microbiota in 2 tilapia sashimi mislabeled as snapper (SN1, HN1: Oreochromis niloticus) and 8 commonly-consumed and correctly labeled sashimi (MT2: Thunnus thynnus; KH1: Atheresthes stomias; FW1: Makaira nigricans; FS1: Salmo salar; KY1: Seriola dumerili; IN1: Pagrus major; UY2: Seriola quinqueradiata; KH2: Cololabis saira). Significant difference of relative abundance of Pseudomonas and Dechloromonas were found between tilapia and commonly-consumed sashimi samples (both *P* = 0.044). (**B**) Rarefaction curves of the 10 samples in the microbiome sequencing saturation analysis.

The above analysis revealed a high percentage of snapper substituted by tilapia, either in sashimi dishes or in cooking materials (Table 1). Snapper has been conventionally served as sashimi for a long time in Japan; Tilapia has not. As sashimi are consumed raw, snapper sashimi substituted by tilapia raises potential health concerns apart from fair-trade concerns. Thus, we conducted a deep microbiome profiling of 10 sashimi samples, including 2 tilapia sashimi (SN1, HN1: *Oreochromis niloticus*) served in two different restaurants, and 8 correctly-labeled conventional Japanese sushi (MT2: *Thunnus thynnus*; KH1: *Atheresthes stomias*; FW1: *Makaira nigricans*; FS1: *Salmo salar*; KY1: *Seriola dumerili*; IN1: *Pagrus major*; UY2: *Seriola quinqueradiata*; KF2: *Cololabis saira*). The relative abundance of the *Pseudomonas* genus is significantly higher in tilapia sashimi (Mann–Whitney test, *P* = 0.044). On the other hand, the abundance of *Dechloromonas* is lower (Mann–Whitney test, *P* = 0.044, Fig. 4A). No significant difference was found in the genera of *Acinetobacter, Alkanindiges, Aquabacterium, Bacillus, Brevibacillus, Psychrobacter, Sphingobium, Sphingomonas*. To evaluate whether the current microbiome sequencing depth is sufficient, we performed sequencing saturation analysis. Rarefaction curves were plotted using random subsets of sequences with different subset sizes, simulating the number of identified species given different sequencing depth (Fig. 4B). When the sequencing depth is small, number of identified species increases quickly. When the sequencing depth approaches the current depth of this study (i.e. > 100,000), the slope is greatly reduced, suggesting that the current sequencing depth is sufficient.

We further scrutinized the sequence reads associated with the *Pseudomonas* genus to investigate the species involved. Four different species were found in tilapia sashimi: *P. reactans, P. oryzihabitans, P. resinovorans* and *P. peli.* The pathogenic *P. aeruginosa* is not identified, rather *P. resinovorans* is found which has been placed in the *P. aeruginosa* group in one taxonomic study^25^. *P. oryzihabitans* has been reported to have caused a life-claiming sepsis in a 5-month old boy^26^. In our data, only *P. peli*. manifested statistical significance between tilapia sashimi and other sushi (Mann–Whitney test, *P* = 0.044). *P. peli.* has recently been found in the drinking water distribution systems and is chorine-resistant^27^. Our initial data suggested that future, larger-scale investigations are warranted.

## Discussion

The sashimi and nigiri sushi are classic Japanese cuisine which have gained global recognition. Being meals served raw, health issues need to be taken into consideration. In this study, we found a high proportion of snapper products substituted by tilapia (*Oreochromis niloticus*), some of which were even labeled as “snapper sashimi” and served raw in restaurants. Unlike conventional Japanese sushi, tilapia sashimi have not been in the dinner table for long. Tilapia mainly live in fresh water^28,29^, while many fish used for Japanese sashimi are ocean dwelling. Tilapia is farmed in outdoor fresh-water ponds, brackish and sea-water ponds in Taiwan. The health impacts of eating raw tilapia has not been studied. Hence, we performed a pilot evaluation of microbiome using the DNA sequencing technology. The result showed that *Pseudomonas* is particularly enriched in tilapia in contrast with conventional Japanese sushi. *Pseudomonas* is a genus of gram-negative, mostly aerobic bacteria commonly live in soil and aquatic habitats^30^. Some strains of *Pseudomonas* is resistant to antibiotics and chorine^27,31^. The notorious member of this genus, *P. aeruginosa*, is responsible for many opportunistic infections in the intensive care units (ICUs) of the hospitals^32^. It may also trigger pseudomonas septicemia. Previously, *P. aeruginosa* was identified in tilapia fresh filets and subsequently cultured successfully^33^. Also, *Pseudomonas* have been found in fresh-water fishes caught by local fishermen in tributaries of the Amazon basin in Brazil (93.3%, 28/30)^34^.

The substitution of snapper by tilapia is actually originated from the confusing practice of labeling Tilapia as Taiwan-Snapper (臺灣鯛), driven mainly by the commercial interests of some local vendors. The annual production of tilapia is more than 70 thousand metric tons, accounting for 100 million USD and 20–25% of total aquaculture values. Being a sizable food industry, many tilapia products are commercially marketed as “Taiwan Snapper” for the benefits of marketing and sales. One of our sample molecularly identified as tilapia has been labeled clearly as “Taiwan Snapper”, and are not considered fraudulent for the time being. Nevertheless, the other 11 tilapia products are labeled as snapper without the prefix of “Taiwan”, and are judged fraudulent in this study. Taxonomically, snapper and tilapia are distinct species. They both belong to the biological Class of *Actinopterygii* but differ in Order (*Perciformes* and *Cichliformes* for snapper and tilapia respectively). Despite the commercial interests, tilapia labeled as “Taiwan snapper” is scientifically incorrect and may cause unnecessary confusion and consequences.

The substitution of cod by halibut is another category of seafood labeling fraud in Taiwan, echoing studies in other places^10,14^. The consequence of this fraud is mainly an unfair trade. Fillets of cod and halibut are similar in appearance. Their difference in taste and texture is also very subtle. According to the governmental regulations of the Food and Drug Administration of Taiwan (TFDA), only the fishes in the biological Order of *Gadiformes* can be labeled as cod (in Chinese: 鱈魚)^35^. The frequent substitution of cod by halibut in Taiwan showed that government oversight should be tightened for ensuring fair trades.

We relied on the GenBank of the US National Center of Biotechnology Information (NCBI), and the Barcode of Life Data System (BOLD)^36,37^ for identifying the biological origins of the food. There are a few occasions when the NCBI and BOLD systems gave different top-match results, largely due to the different reference sequences contained in these systems. Different top matches can be broadly classified as the following scenarios. The first scenario involved two samples which were identified as *Makaira nigricans* (Atlantic blue marlin) by NCBI and *Istiompax indica* (black marlin) by BOLD. Marlins/billfishes both belong to the biological Order of *Istiophoriformes*. In the local food industry, these billfishes are sold under the same umbrella name in Chinese (旗魚, translated as swordfish). As these two products were labeled by their common name encompassing *Makaira nigricans* and *Istiompax indica*, we judged that they are correctly labeled. The second scenario involved a sample labeled as cod and molecularly identified as *Epinephelus diacanthus* by NCBI and *Priacanthus hamrur* by BOLD. Neither of the two identified species belong to the biological Order of *Gadiformes*, the official definition of cod in Taiwan, therefore, we judged this product mislabeled. The third scenario occurred when one system gave a species-level information (e.g. *Oreochromis niloticus* and *Thunnus atlanticus*) while the other system gave only a genus-level information (e.g. *Oreochromis sp.* and *Thunnus sp*.). In these cases, the genus level information is often sufficient to judge the products sold by their common names. The fourth scenario occurred at three samples where the NCBI gave species-level identification while the BOLD showed no match. In these cases, we relied on NCBI alone to make the judgments.

We also found that surimi product names are often inconsistent with their molecularly identified ingredients. The surimi products are often labeled as if they were made by a major seafood ingredient, for example, “big lobster sticks” and “chewy cod balls”. As a matter of fact, they are named arbitrarily and creatively by their resemblance in shapes and colors with other more appealing seafood ingredients (e.g. lobster and cod). The products were actually made by the mixture of some other fishes (e.g. groupers) and non-fish ingredients. Unfortunately, most consumers are not aware of the inconsistency. This showed a currently under-regulated seafood category in Taiwan which may be mitigated by more stringent government regulation with the availability of DNA barcoding technology.

The same metagenomics approach in this food-related study has been used previously in other environment or biological studies^38,39^. For example, the microbiome of the fish intestine and gill has been investigated^9^. The sashimi samples in the current study were basically fish meat. We assumed that when the fishes were no longer alive, the immunity of the fish subside and the microbes would be able to spread from their originally enriched site to other parts of the body. We cannot, however, rule out completely the possibility of restaurant contaminations in the current pilot study. Decoupling the microbes from the fish and from the restaurants requires larger, deep microbiome investigations in multiple types of sashimi samples collected from the same restaurants, and the same fish type in multiple restaurants. This remained our future research.

In conclusion, the gross seafood mislabel rate in Taiwan is 18.9% [12.5–26.8%]. Snapper, cod and surimi products are particularly vulnerable to fraudulent substitutions. Our pilot microbiome study showed that Tilapia sashimi fraudulently labeled as snapper might have higher levels of *Pseudomonas* than conventional Japanese sashimi, raising health concerns in addition to trade fairness issues. This however requires future, larger studies to confirm.

## Method

### Study sample preparations

This is a consumer-centered survey that a total of 127 seafood samples were purchased by designated customers with minimal disruption to the routine commercial activities in the restaurants and retail markets in Taiwan. The labels of all these products were photographed for records (exemplified in Fig. 2). Samples were brought to the laboratory and dissected into small pieces, transferred into 1.5 mL microcentrifuge tubes, and stored in a − 30 °C refrigerator. For meals comprising both seafood and non-seafood ingredients, we only collected the seafood part as study samples. For example, we examined only the raw fish meat of the nigiri sushi and excluded the accompanied vinegar-flavored rice.

Samples were then used for DNA extractions. 5–10 g of seafood tissue samples were thawed and grinded with scalpel. DNA was extracted from 25 mg of grinded tissue. Samples were treated with lysozyme and followed by 15 h of proteinase K treatments. The extracted DNA were then used separately for fish species identification and the microbiome metagenomics profiling.

### Reviewing fish-species identification primers in literature

**Figure 5:**
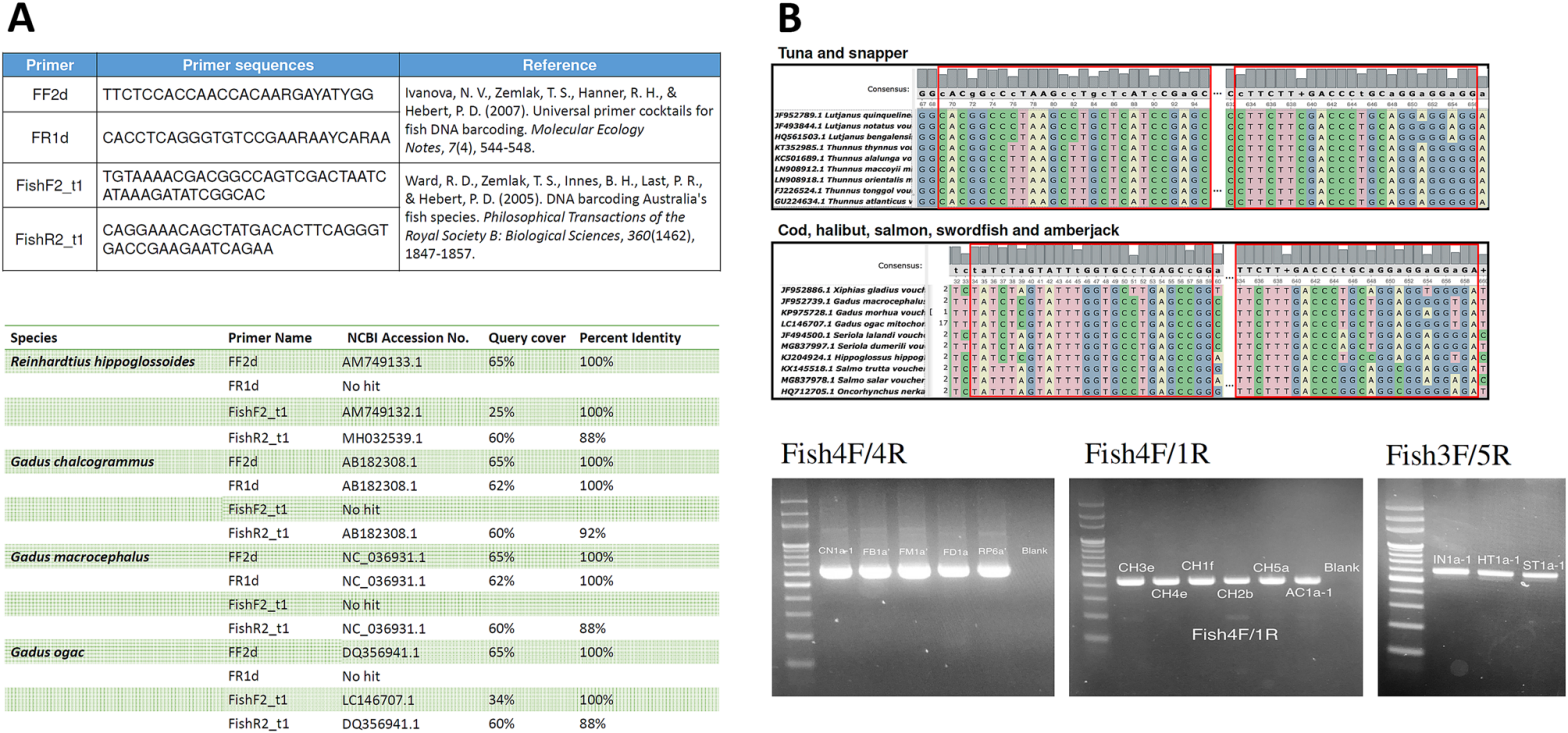
Primers for the fish taxonomic identification. (**A**) The primers in literature were aligned to reference sequences of *Reinhardtius hippoglossoides, Gadus chalcogrammus, Gadus macrocephalus* and *Gadus ogacin* in NCBI. These primers manifest poor query coverage (< 70%) to the fish species of interest in this study. (**B**) Primers and gel images of the amplified DNA. The primers Fish1R and Fish4F/4R were designed using the multiple sequence alignment of the reference sequences of cod, halibut, salmon, swordfish and amberjack. Fish3F and Fish5R were based on tuna and snapper.

The mitochondrial cytochrome c oxidase subunit I (MT-CO1) DNA sequences were selected for fish taxonomic identification. We first examined the previously published primers in literature^40,41^ to see whether they can be used for the identification of the fish of interest in this study. The BLAST alignment online tool was used to match these primer sequences against the reference sequences in the NCBI GenBank. The primers manifest poor query coverage (< 70%) in general, sometimes accompanied by low percent identity (< 90%) with the reference sequences of cod and halibut (Fig. 5A) as well as tuna, salmon, swordfish, snapper and amberjack. The low query coverage indicated that they may not be able to anneal properly with the DNA of the fish of interest in the present investigation.

### Amplification and sequencing of the MT-CO1 gene for fish taxonomic identification

New primers were designed using the conserved regions, found via the multiple sequence alignment of the reference sequences in the NCBI GenBank, using the bioinformatics software UGENE v1.31.1 (Fig. 5B). We designed two forward primers Fish3F, Fish4F and three reverse primers Figh1R, Fish4R, Fish 5R (sequences shown in Table 2**)**, for coping with a wide diversity of fish species which may have small nucleotide differences in the primer regions. Fish1R and Fish4F/4R were based on the reference sequence of cod, halibut, salmon, swordfish and amberjack; while Fish3F and Fish5R were based on tuna and snapper. The amplicon sizes ranged between 520–625 nucleotide bases. These primers all have adequate query coverage (100%) and percent identity (> 90%) in the aforementioned fish types.

**Table 2:** Primers used for MT-CO1 DNA polymerase chain reaction and sequencing.

Primer name	Sequence
Fish1R	TTAATTGCCCCAAGAATTGATGAAAT
Fish3F	CACGCCTTAAGCTTGCTCATCCGAGC
Fish4F	TATCTAGTATTTGGTGCCTGAGCCGG
Fish4R	TCACCTCCTCCAGCAGGGTCAAAGAA
Fish5R	TCCCCTCCGCCTGCCGGGTCAAAGAA

F: forward primer. R: reverse primer. The direction of F and R is based on the MT-CO1 gene direction.

DNA (200 ng/sample) extracted from the fish samples were then processed by polymerase chain reaction (PCR). The initial denaturation temperature of the PCR process was 95 °C for a duration of 1 min. The denaturation, annealing and extension temperatures were as 95°, 60° and 72° respectively, each proceeded for 30s. The above procedure was iterated for 35 cycles. Final extension was performed at 72 °C for 5 min. The extracted and amplified DNA was then checked using electrophoresis, together with negative controls (water). Electrophoresis was used to check whether the amplified DNA has the expected size. Also, the DNA spectrometer was used for checking the DNA quality. Samples with the A260/280 ratios between 1.75 and 1.95 were accepted. Sanger sequencing were performed on the amplified DNA, using both the forward and reverse primers. The primer sequences were trimmed away from the sequence reads. The sequence reads of the samples were searched against the references in the NCBI GenBank and BOLD^36,37^ for molecular taxonomic identification.

### Microbiome profiling of sashimi samples based on the 16S rRNA genomic segments

The hypervariable regions V3 and V4 of the 16S ribosomal RNA (16S rRNA) gene^42^ were amplified using the adapter-incorporated primers for microbiome studies. Forward: 5′-**TCGTCGGCAGCGTCAGATGTGTATAAGAGACAG***CCTACGGGNGGCWGCAG*-3′, and Reverse: 5′-**GTCTCGTGGGCTCGGAGATGTGTATAAGAGACAG***GACTACHVGGGTATCTAATCC*-3′. The adapters (shown in bold) were designed by Illumina, the next generation sequencing platform provider. The locus-specific primers (shown in *italic face* and underline) were selected for amplifying the 16S V3 and V4 region of *Bacteria* and *Archaea* optimally^43^. The amplicon size is ~ 428 bases, depending on the microbe. DNA (2.0 µg/sample) extracted from the fish samples were then processed by the sequencing via synthesis using the Illimina sequencing platforms and the MiSeq v3 sequencing chemistry, with more than 100,000 sequence reads per a sample. Paired-end reads were merged using the FLASH bioinformatics tool^44^, de-multiplexed, adaptor-incorporated primers trimmed, chimera removed using the UCHIME bioinformatics tool^45^, and quality-filtered to produce effective tags. The statistics of ultra-deep amplicon sequencing for microbiome profiling is summarized in Table 3.

**Table 3:** The statistics of ultra-deep amplicon sequencing for microbiome profiling.

Sample ID	Raw pair-end number	Raw tags	Clean tags	Effective tags	Average length of effective tags	Q20	Q30	Effective rate (%)
FS1	153,422	120,793	112,796	112,478	459	99.36	97.1	73.31
FW1	181,014	157,351	152,099	148,456	461	99.41	97.22	82.01
HN1	216,086	194,684	187,430	182,433	463	99.39	97.36	84.43
IN1	208,013	188,828	178,386	176,822	451	99.45	97.52	85.01
KF2	199,342	177,006	170,810	165,564	460	99.39	97.24	83.06
KH1	145,079	130,261	126,373	124,783	457	99.4	97.3	86.01
KY1	228,048	203,266	196,470	191,498	457	99.4	97.32	83.97
MT2	244,559	215,409	206,096	205,420	456	99.4	97.27	84
SN1	190,400	167,395	162,765	160,361	459	99.45	97.43	84.22
UY2	212,253	183,010	176,131	174,688	460	99.34	97.02	82.3

Raw pair-end number are the number of total pair-end reads derived from the samples. Raw Tags are the tags merging the pair-end reads. Clean Tags are the remaining tags after low-quality and unusually short tags are removed. Effective Tags are the remaining tags after the chimera are removed. Q20 and Q30 refer to the percentage of bases with individual Q values greater than 20 (representing the sequencing error rate less than 1%) and 30 (error rate less than 0.1%) in effective tags. Effective rate (%) is the ratio of the number of effective tags and the number of Raw pair-end reads.

The effective tags were then used for microbiome taxonomic identification. We first clustered these effective tags with > 97% percent identity into operational taxonomic units (OTUs)^46^ using the UPAIRSE algorithm^47^ in the USEARCH bioinformatics package (v 7.0). The identified OTUs were then annotated using the ribosomal database project (RDP) Classifier^48^, a naïve Bayesian approach offering taxonomic assignments from the Domain level to the Genus level. Additional detailed annotation was obtained by sequence alignments with the microbiome references in the NCBI GenBank.

### Statstical analysis

The non-parametric Mann–Whitney tests were used to compare the relative abundance of the microbe genus. The confidence intervals of the substitution rates were calculated using the exact binominal method provided by the UCSF online calculator (http://sample-size.net/confidence-interval-proportion/).

## Data Availability

We planned but have yet to construct a designated online database for hosting the raw data of seafood authentication and microbiota profiling, together with user-friendly data browsing tools. In the meantime, raw data are available from the corresponding authors (T.Y.L. and K.H.L) on reasonable requests from academic scientists.
